# Exploration of the Fluorescent Properties and the Modulated Activities against Sirtuin Fluorogenic Assays of Chromenone-Derived Natural Products

**DOI:** 10.3390/molecules23051063

**Published:** 2018-05-02

**Authors:** Hui Wen, Nina Xue, Feng Wu, Yujun He, Gang Zhang, Zebin Hu, Huaqing Cui

**Affiliations:** 1 State Key Laboratory of Bioactive Substances and Function of Natural Medicine, Institute of Materia Medica, Peking Union Medical College and Chinese Academy of Medical Sciences, Beijing 100050, China; wenhui@imm.ac.cn (H.W.); angelnina@imm.ac.cn (N.X.); heyujun506@imm.ac.cn (Y.H.); gzhang@imm.ac.cn (G.Z.); 2 Institute of Aviation Medicine, Air Force, Beijing 100142, China; wufeng879@126.com; 3 National Institutes for Food and Drug Control, Beijing 100050, China

**Keywords:** chromenone, isoflavone, osthole, AMC, fluorescence, fluorogenic assay, sirtuin, SIRT1 activator

## Abstract

Chromenone-derived natural products include chromones (flavone, isoflavone) and coumarins. Chromenone compounds not only exhibit impressive biological activities, but also are an important resource of experimentally used fluorophores, such as, 7-amino-4-methylcoumarin (AMC). Various chromenone compounds have reported to have weak fluorescence, and this has the potential to interfere with the measurements during AMC fluorogenic assays and result in non-robust assay readouts. Several flavones and isoflavones were found as SIRT1 activators, while fluorogenic sirtuin assays utilized AMC labelled peptides as the substrates. In this study we investigated whether the fluorescent properties of chromenone-derived natural products interrupt the measurement of SIRT1/2 modulated activities. We found that the reported SIRT1 activators: flavones were detected with the SIRT1 activation activity, but isoflavones were not detected with SIRT1 activation activity, and instead that they were found to be fluorogenic compounds. Another chromenone compound, osthole, exhibited a moderate SIRT2 inhibitory activity with an IC_50_ of 10 μM. In conclusion, the fluorescent properties of these chromenone compounds do affect the measurement of the sirtuin activities of both inhibitors and activators. However, if the possible fluorescence properties are mitigated in the assay readout, these fluorogenic assays enable the screening of activity modulators.

## 1. Introduction

**Figure 1:**
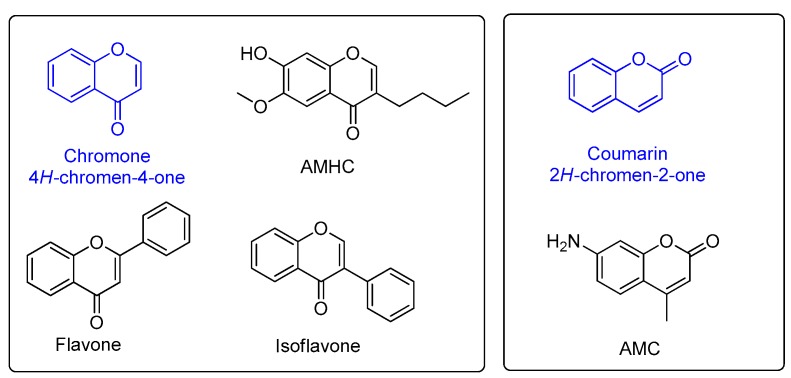
Chemical structures of chromenone derived compounds (chromone and coumarin). The left handside shows the structures of chromone and other chromone derived compounds, such as, flavones, isoflavone and 3-alkyl-6-methoxy-7-hydroxychromone (AMHC). The right handside shows the structures of coumarin and 7-amino-4-methylcoumarin (AMC).

Flavonoids are a large family of natural products, which are mainly found and extracted from plants [1,2,3]. Chromenone-derived compounds belonging to the flavonoid family include chromones (flavones, isoflavones) and coumarins (Figure 1) [4,5]. Chromenone is recognized as a privileged scaffold in medicinal chemistry due to its advantageous drug-like properties and versatile binding properties [4,5]. Various chemical synthesis and modification of the structure of chromenones (chromones and coumarins) have been extensively studied and numerous pharmacological activities of these important scaffold have been revealed, such as, anti-inflammatory [6], anti-oxidant activity [5], cardiovascular and hepatic protection effects [4].

Interestingly, many chromenone-derived compounds were found to be fluorogenic [7,8], with the 7-hydroxycoumarin scaffold being used as the main experimental chromenone fluorophore [9,10]. Commercially available 7-amino-4-methylcoumarin (AMC) is commonly used in various biological studies, and in particular AMC was used to develop a fluorescent turn-on enzymatic assays to study a large range of proteins including histone deacetylase (HDAC) [11], sirtuin (SIRT) [12], amidase [13], transglycosylase [10], and alkaline phosphatase (ALP) [14]. 7-Hydroxyisoflavones and 3-hydroxyflavones also exhibit fluorescence in biological buffers [15,16]. In our previous studies, we reported the development of 3-alkyl-6-methoxy-7-hydroxychromones (AMHC) as a fluorogenic scaffold for biological studies, which displays comparable fluorescent properties to AMC [7]. Notably, both AMC and AMHC are chromenone-derived compounds, and they have similar excitation and emission wavelengths (*E*_x_ = 360 nm, *E*_m_ = 460 nm) as other fluorogenic chromenone compounds.

The sirtuin family of enzymes are a class III histone deacetylase (HDAC) that catalyzes the removal of acetyl groups from a lysine residue found on endogenous substrates [17,18]. The sirtuin family includes seven enzymes, however SIRT1 and SIRT2 are the most well studied members of this family. SIRT1 involves cell metabolism, and regulates fat and cholesterol metabolism, with SIRT1 deficiency in mice resulting in insulin resistance [19,20]. SIRT2 functions in the cellular activities, such as, necrosis and apoptosis. Genetic knockout of SIRT2 and small molecule SIRT2 inhibitors both show neuroprotection against the neurotoxicity observed in a Parkinson’s disease cell model and a transgenic mouse model [12,21,22].

**Figure 2:**
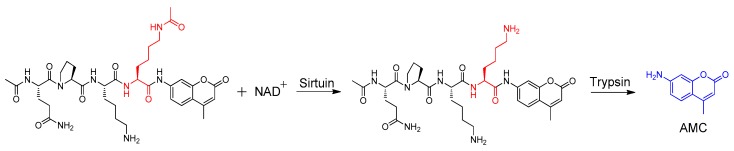
The principle of AMC labelled fluorescence turn-on sirtuin assay. The AMC labelled peptide containing an acetylated lysine, in which the fluorescence is quenched. This peptide was used as the substrates in the assay and can be deacetylated by sirtuin enzyme in the presence of NAD^+^. The deacetylated peptide is then digested by trypsin to release the free AMC, which has turn-on fluorescence. Thus, the measured fluorescence intensity can be used as a readout for the activity of the sirtuin enzyme.

Due to their importance, sirtuin enzymatic assays have been developed employing different strategies [23,24,25,26,27,28], such as, using FRET assays and fluorescent turn-on assays. However, the most commonly used sirtuin enzymatic assay were developed using AMC-labelled peptide serving as the substrate, in which the lysine was acetylated (Figure 2) [12,27]. Once the peptide was deacetylated by the sirtuin enzyme, the peptide was digested by trypsin to release the fluorescent tag and quantified. In this assay, 7-amino-4-methylcoumarin (AMC) was used as the fluorophore.

**Figure 3:**
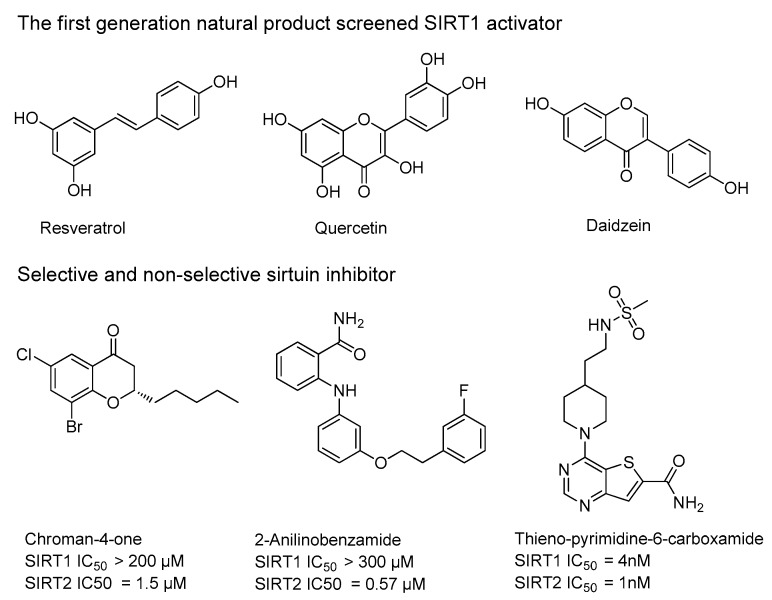
The structures of selected SIRT1 activators and SIRT1/2 inhibitors. The first generation SIRT1 activators were discovered from screens of flavonoids natural products. Resveratrol is one of the most potent SIRT1 activators discovered so far, while flavones (quercetin) and isoflavones (daidzein) were also found with SIRT1 activation activity. For sirtuin inhibitors, many synthesized compounds were reported with selective or non-selective sirtuin inhibitory activities.

Many sirtuin modulators have been discovered, which include SIRT1 activators and various sirtuin inhibitors (Figure 3) [12,29,30,31,32,33,34,35,36,37,38]. SIRT1 activators were initially screened from flavonoid-derived natural products. Among the first generation of naturally sourced SIRT1 activators, resveratrol exhibited the best SIRT1 activation activity and it was shown to be able to extend the lifespan of yeast [33]. Notably, several flavones and isoflavones were also reported as SIRT1 activators [33,36]. The pharmacological effects of SIRT1 activators have been observed in animal models with remarkable health benefits, although the mechanism of action of these SIRT1 activators is still not established [39]. On the other hand, many compounds have been developed as sirtuin inhibitors [12,29]. Among them, some of them were designed as selective inhibitors against a certain member of sirtuin family, while others exhibited pan-inhibition of the entire family. Notably, a series of chromenone compounds were found to be potent SIRT2 selective inhibitors [30].

Sirtuin fluorogenic assays were developed using AMC as the fluorescent tag. Considering the similar fluorescence emission and excitation wavelengths of AMC and some fluorogenic chromenone compounds, in this study we investigated the fluorescence properties and sirtuin modulated activities of chromenone compounds in order to investigate how the fluorescence properties can affect the measurement of fluorogenic enzymatic activities of chromenone compounds.

## 2. Results and Discussion

### 2.1. The Fluorescence Properties of Chromenone Derived Natural Products

A group of chromenone derived natural products (Table 1), including flavones, isoflavones and coumarins, were collected and initially screened for fluorescent properties. The maximum excitation wavelength, emission wavelength and the fluorescent quantum yield of these compounds were measured in aqueous PBS buffer (pH 7.4). We found that several naturally derived chromenone compounds: formononetin, daidzein and calycosin showed clear fluorescence in PBS buffer, with the maximum excitation and emission wavelengths in the range of *E*_x_ (338–348 nm) and *E*_m_ (478–490 nm), which is close to that of AMC (*E*_x_ = 360 nm, *E*_m_ = 460 nm). Another flavonoid compound, resveratrol, is also fluorogenic, and has *E*_x_ = 304 nm, *E*_m_ = 400 nm, which differs slightly from AMC. The remaining compounds exhibited no or marginal fluorescence in PBS buffer.

Natural products are a major chemical resource used for hit finding in drug discovery. Interestingly, many natural products are fluorogenic compounds, however they usually have relatively low fluorescent quantum yields. Several commonly used experimental fluorophores with enhanced fluorescence properties were modified from the fluorogenic natural product [7]. In this study, we chose natural products as the targeted samples. For compound selection, we included several previously reported chromenone derived SIRT1 activators [33,36], such as, flavone compounds (quercetin, luteolin, kaempferol), isoflavone compounds (daidzein) and other flavonoids (resveratrol). This should allow for a better comparison of the fluorescent properties and SIRT1/2 modulation activities of this class of compounds. For the examination of fluorescence properties, we detected and recorded the maximum *E*_x_ and *E*_m_, which will be used to compare with that of AMC. We also measured and compared the fluorescence quantum yields of chromenone compounds (around 0.05 in PBS buffer) [7]. The excitation and emission fluorescence spectra of the fluorescent compounds were included in the supplementary file. Interestingly, three previously reported flavone derived SIRT1 activators (quercetin, luteolin, kaempferol) are all non-fluorogenic compounds in PBS buffer. However, we observed that the isoflavone derived SIRT1 activator daidzein and the flavonoid compound resveratrol are fluorogenic compounds.

### 2.2. The SIRT1/2 Activities of Chromenone Derived Natural Products

Next, we screened the SIRT1/2 activity of chromenone compounds (Table 1). All compounds were dissolved to a final screening concentration of 10 μM. For the SIRT1 activities, we found that resveratrol showed potent active SIRT1 activation (activation rate = 11.6). Flavone compounds (quercetin, luteolin, kaempferol) also displayed low activation activity, but for the remaining flavone compounds, we did not observe any obvious activation activity toward SIRT1. No isoflavone compound with SIRT1 activation activities was detected, although some of them, such as, daidzein, were previously reported to have SIRT1 activation activities. However, baicalin showed weak SIRT1 inhibitory activity. For SIRT2 activities, no compound tested displayed any SIRT2 activation activity. Most of the compounds tested were found to have weak SIRT2 inhibitory activities. However, osthole was found to have moderate SIRT2 inhibitor (47% enzymatic activity at the concentration of 10 μM).

Sirtuin fluorogenic assays have been developed utilizing various AMC labelled peptides as the substrate, with this assay being routinely used to screen for small molecule SIRT1/2 modulators [27]. The first generation of SIRT1 activators were screened using the commercial Fluor de lys assay kit (ENZO Company, New York, NY, USA, BML-AK500-0001, *E*_x_ = 360 nm, *E*_m_ = 460 nm), which was initially developed as a HDAC assay and included two amino acid AMC labeled peptide as the substrate [28]. However, currently various 4-mer peptides are more often used in these sirtuin assays. It is clear that different peptide substrates have differing *K*_m_ in sirtuin assay, and this will subsequently affect the detected activity of various modulators. Therefore in this study we only observed weak or marginal SIRT1 activation activity for the flavones compounds (quercetin, luteolin, kaempferol), although these were previously reported as having potent SIRT1 activation activities. However, and in accordance to previously published work, resveratrol still acted as very potent SIRT1 activator in our study. Unfortunately, the isoflavones derived compounds did not show any activation activity toward SIRT1 even though they were reported as having clear SIRT1 activation activity [33,34,38].

The fluorophore of AMC was used in our sirtuin fluorogenic assays, with detection carried out at specific wavelengths (*E*_x_ = 360 nm, *E*_m_ = 460 nm) [12]. Due to the similar excitation and emission wavelengths of fluorogenic chromenone compounds with AMC (Table 1) [7], we reasoned that the observed fluorescence from chromenone compounds might cause the false evaluation of these sirtuin modulators. In particular we were concerned that the final fluorescence turn-on rate was partially of the substrate peptide (50 μM). In comparison, the concentration of screened compounds was usually set with 10–50 μM. Thus, possible fluorogenic compounds might cause the fake activation but hide the potent inhibition.

In this study, which contained compounds with known fluorescence included resveratrol and several isoflavones derived compounds, we removed the fluorescent background from the assay readout to calculate the enzymatic activity (Table 1). Our results showed that several flavones compounds (quercetin, luteolin, kaempferol) are non-fluorogenic and acted as SIRT1 activators and SIRT2 inhibitors. However, the isoflavone compounds (formononetin, calycosin, glycitein) are reasonably fluorogenic. If the fluorescent signals of these compounds were removed, we observed that they exhibited weak SIRT1 and SIRT2 inhibitory activities instead of being as SIRT1 activators. Thus, the possible fluorescence properties of these chromenone compounds do indeed interrupt and interfere with the regular performance of AMC applied sirtuin fluorogenic assay, and thus affects the robustness of the readout obtained.

### 2.3. The Inhibitory Mode of Osthole against SIRT2

**Figure 4:**
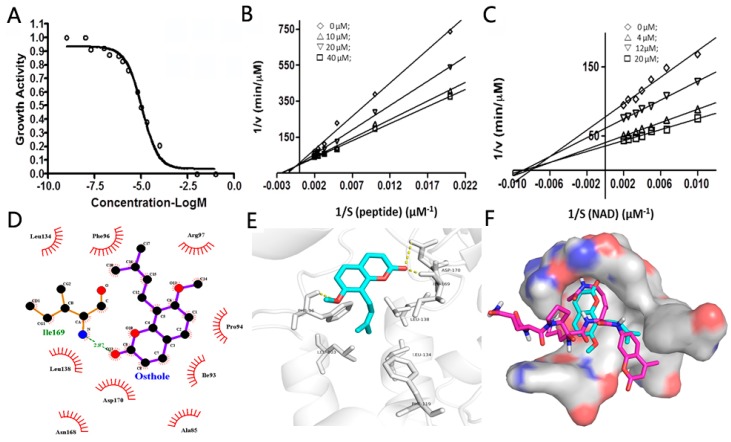
The inhibition mode study of osthole against SIRT2. (**A**) The measurement of IC_50_ of osthole against SIRT2. Osthole was used from 0, 0.05, 0.1, 0.16, 0.5, 0.63, 1, 5, 10, 50, 100 µM. (**B**) The peptide substrate was used at various concentrations (0, 50, 100, 200, 300, 400 and 500 µM) and osthole was used at 0 (◊), 10 µM (△), 20 µM (▽), 40 µM (□) with NAD^+^ held at 0.5 mM. (**C**) NAD^+^ was used at the concentrations (0, 100, 150, 200, 250, 300, 400 and 500 µM) and osthole was used at 0 (◊), 10 µM (△), 20 µM (▽), 40 µM (□) with the peptide substrate held at 0.35 mM. (**D**,**E**) Docking study of osthole binding in the active center of SIRT2. (**F**) The docking study shows that osthole occupy the space of peptide substrates in the active center of SIRT2. The cyan compound represent osthole, the purple compound is the peptide substrate. Accelrys discovery studio visualizer 4.0 and Pymol 0.99 were used for the studied, and crystal structure of SIRT2 was selected as PDB Code: 1j8f.

We applied the aforementioned AMC used SIRT1/2 fluorogenic assay to screen a series of chromenone-derived compounds. This screen identified several compounds with appreciable activity, however, we decided to focus our attention on osthole, which showed moderate SIRT2 inhibitory activity (Table 1). In order to understand the *bona fide* inhibitory mode of osthole against SIRT2, we performed the enzymatic inhibitory assay with different concentrations of osthole, and found that osthole showed potent SIRT2 inhibition activity with an IC_50_ of 10 µM (Figure 4A). As there are two substrates, NAD^+^ and the peptide, used in the assay, we decided to investigate the competitive relationship between substrate peptide and osthole. We saturated the system with NAD^+^, and then varied the concentration of the peptide used. We measured the enzymatic kinetics at different inhibitor concentrations, and plotted the graph of 1/rate (v) versus 1/peptide with differing inhibitor concentrations. We observed that osthole was a competitive against the substrate peptide (Figure 4B). We then turned our attention on NAD^+^ competition and saturated the assay with high concentration of peptide and subsequently added various concentrations of NAD^+^ to measure the enzymatic kinetics at each inhibitor concentration, and plotted the 1/rate (v) versus 1/NAD^+^ . The results show that osthole is a non-competitive inhibitor against NAD^+^ (Figure 4C). The mode of action study of osthole against the substrates of SIRT2 assays suggests osthole competes with the substrate peptide binding events at the peptide-binding site within the SIRT2 active site, but does not affect the binding of NAD^+^ to the enzyme.

We next wanted to use in silico methods to understand the binding mode of osthole inside the active site of SIRT2 [40]. For the docking studies, the Accelrys Discovery Studio Visualizer 4.0 (Accelrys, San Diego, CA, USA) and Pymol 0.99 was utilized for interaction visualization, and X-ray crystallographic structure of SIRT2 (PDB Code: 1j8f) were obtained from the protein databank (PDB). The lowest energy conformations were selected and the ligand interactions with SIRT2 were determined. These calculations showed that osthole formed hydrogen bond interactions with N–H of ILE169 residue, O–H of ASP170 and N–H of PHE96, respectively. The phenyl ring of osthole showed weak π-π interaction with the phenyl ring of PHE96. The alkene chain of osthole showed hydrophobic or Van der Waals interactions with the hydrophobic groups of LEU103, PHE119, LEU134 and LEU138 (Figure 4D–E). In addition, the docking study also showed that osthole shared the same binding pocket with the substrate peptide, which occupied the lysine position on C-terminal of the substrate peptide (Figure 4F).

As we summarized above, the fluorescence properties of chromenone compounds do affect the measurement of AMC applied fluorogenic assays through fluorescence interference. In this study, our SIRT1/2 fluorogenic assay showed that resveratrol was a potent SIRT1 activator and osthole as a moderate SIRT2 inhibitor. The activation mechanism of resveratrol toward SIRT1 has been intensively studied by many research groups although the underlying mechanism is still under investigation [36,37,39]. In order to more thoroughly understand the mode of inhibition of osthole against SIRT2, we decided to characterize the enzymatic inhibition mechanism of ostholes against SIRT2. Osthole competes with the peptide substrate within the peptide binding site, but does not bind to NAD^+^ binding area. Particularly, computation methods also revealed osthole occupied the space of the peptide substrate, which agreed the kinetic study. All of the above data prove the *bona fide* inhibition of osthole against SIRT2. Thus, if the possible fluorescence properties of chromenone compounds were taken into consideration and therefore mitigated, AMC applied fluorogenic assays enable the screening of *bona fide* enzyme modulators.

## 3. Materials and Methods

### 3.1. The Chromenone Derived Natural Products

All chromenone compounds with the purity of more than 95% were purchased from the Beijing Shiji-Aoke Biotechnology Co. (Beijing, China) and the Shanghai Jingke Chemistry Technology Co. (Shanghai, China).

### 3.2. Comparison of Fluorescent Properties of Chromenone Derived Natural Products

To obtain fluorescence excitation and emission spectra, we dissolved compounds in PBS (pH 7.4) at a concentration of 10 μM. For all compounds, we first set the λ_em_ to get the maximum excitation wavelength, we then set λ_ex_ to obtain the maximum emission wavelength [9,41,42,43].

The calculation of fluorescent quantum yield. Fluorescence quantum yield was recorded using a comparative method of Williams et al. [44] The detection was carried out in PBS buffer (pH 7.4) at the concentration of 0.5 µg/mL using quinine sulfate (0.5 µg/mL in 0.1 M H_2_SO_4_, Ф = 0.54) as a reference. The quantum yield was calculated using the following equation:

Ф_X_ = Ф_ST_ (*A_ST_F_x_*/*A_X_F_ST_*)(*n_X_*/*n_ST_*)^2^

where the subscripts *X* and *ST* denote test and standard respectively, Ф is the fluorescence quantum yield, *A* is the absorbance at the excitation wavelength, *F* is the area under the emission curve, and *n* is the refractive index of the solvents used. For the tested compounds and the standard, the excitation wavelength was at 345 nm while keeping the absorption below 0.05.

### 3.3. The Purification of SIRT1 and SIRT2 Recombinant Proteins

The ORF length of human SIRT1 (part) and SIRT2 (whole) was cloned into the expression vector pET28a [12,27,32]. The primers of SIRT1 were: 5′-tatggatccatggcggacgaggcggccctcgccctt-3′, and 5′-tatgcggccgcactatgatttgtttgatggatagt-3′; The primers of SIRT2 were: 5′-tatggatccatggcagagccagacccctctcaccctctgga-3′, and 5′-tatgcggccgcatcactggggtttctccctctctgtt-3′. The plasmids were separately transformed into *E. coli* BL21 (DE3) cells, and cultured the *E. coli* to be OD600 of 0.6, the *E. coli* was induced with 1 mM isopropyl β-d-thiogalactoside (IPTG) and cultured for 6 h at 18 °C. The cultures were harvested by centrifugation and the cell pellets were stored at −20 °C. The cell pellets were suspended in 15 mL binding buffer (50 mM Tris-HCl pH 8.0, 300 mM NaCl). Cells were sonicated on ice for 5 min and centrifuged at 10,000 rpm for 20 min at 4 °C. The recombinant proteins were included in the supernatant. The clear supernatant was then applied to a Ni^2+^ NTA-agarose matrix (Qiagen, Hilden, Germany) column equilibrated with binding buffer (50 mM Tris-HCl pH 8.0, 300 mM NaCl). The column was washed using the binding buffer to remove all of the unbound proteins. Then the bound proteins were eluted with a linear gradient of 0–200 mM imidazole. Fractions containing recombinant protein were collected, concentrated and stored in the buffer (50 mM Tris-HCl, pH 8.0, 265 mM NaCl, 0.2 mM DTT, and 10% glycerol) at −20 °C.

### 3.4. Sirtuin Inhibition Assay

SIRT1/2 enzymatic assay was performed using the substrate peptide of Ac-Gln-Pro-Lys-{Lys-(Ac)}-AMC [12]. The assay was determined in 60 µL assay buffer (25 mM Tris-HCl, pH 8.0, 137 mM NaCl, 2.7 mM KCl, and 1 mM MgCl_2_ and 1 mg/mL BSA), 500 µM NAD^+^, 50 µM peptide substrate and 1.0 µg recombinant SIRT1 or SIRT2 protein. Various concentrations of screened compounds were added into the enzymatic assay (volume < 0.3 µL). The assay was incubated at 37 °C for 2 h. To each well was added another 60 µL assay buffer (50 mM Tris-HCl, pH 8.0, 100 mM NaCl), which contains 10 µL trypsin (60,000 BAEE/mL) and 2 µL SIRT2 inhibitor nicotinamide (120 mM). The reaction were mixed and incubated at 37 °C for 20 min. The fluorescence was measured on a plate reader with excitation at 360 nm and emission at 460 nm. The IC_50_ calculation of SIRT2 inhibitor was calculated using the GraphPad Prism software (5, GraphPad Software Inc., La Jolla, CA, USA). For each concentration of osthole (0, 0.05, 0.1, 0.16, 0.5, 0.63, 1, 5, 10, 50, 100 µM), at least two wells were performed to calculate the average parameter. For these fluorogenic compounds, the fluorescence background from the screened compounds were specially detected and removed from the final calculation of the fluorescence intensities.

### 3.5. The Inhibition Mode Study of Osthole against SIRT2

For the inhibition mode of osthole against SIRT2, the assay was carried out with the condition listed above, but the concentrations of two substrates (NAD^+^ and peptide substrate) were varied [12]. For the inhibition mode against peptide substrate, 500 µM NAD^+^ and various concentrations of peptide substrates (0, 50, 100, 200, 300, 400 and 500 µM) were used in the assay. Under this assay conditions, different concentrations of osthole (0, 10, 20, 40 µM) were added into SIRT2 assay to study the effect of these inhibitors toward to the assignment of the assay. For each condition, 2 wells of assays were performed. The enzyme kinetics were measured, calculated and reflected by the graph of 1/v versus 1/peptide. For the inhibition mode against NAD^+^, 350 µM peptide and various concentrations of NAD^+^ (0, 100, 150, 200, 250, 300, 400 and 500 µM) were used in the assay. Under this assay conditions, various concentrations of osthole (0, 10, 20, 40 µM) were added into SIRT2 assay to study the effect of these inhibitors toward to the assignment of the assay. For each conditions, 2 wells of assays were performed. The enzyme kinetics were measured, calculated and reflected by the graph of 1/v versus 1/NAD^+^ [12]. The inhibition mode were calculated by fitting the data obtained at different substrate of peptide or NAD^+^ under various concentrations of inhibitors to the Lineweaver-Buck double reciprocal plot:

1/V_0_ = (*K*_m_/V_max_) × (1/[S]) + 1/V_max_

### 3.6. The Docking Study of Osthole Binding to SIRT2

For the docking studies, the Accelrys Discovery Studio Visualizer 4.0 and Pymol 0.99 was utilized for interaction visualization [40], and X-ray crystallographic structure of SIRT2 (PDB Code:1j8f) was obtained from the protein databank (PDB) [22]. PDB protein and molecules were prepared by adding hydrogen, adding missing residues, and converting to pdbqt format. Molecule was docked using Vina with exhaustiveness grade 8, with up to 9 poses saved per molecule. The docking procedure was carried out for the unchanged conformation of the receptor and flexible ligand molecules.

## 4. Conclusions

Chromenone derived compounds are a class of privileged scaffolds within medicinal chemistry, with many chromenone compounds exhibiting impressive biological activities. Interestingly, chromenone compounds are also the main sources for experimental fluorophores. Several chromenone compounds have been well recognized as commercial fluorophores, such as AMC and AMHC. In order to accurately define the modulation activities of chromenone compounds when screened in AMC fluorogenic assays, we investigated SIRT1/2 modulated activities and fluorogenic properties of chromenone-derived natural products. We found that the fluorescent properties of chromenone-derived natural products do indeed affect the performance of AMC–detected sirtuin activity assays. However, if the possible fluorescence properties of chromenone compounds are mitigated and cancelled in the assay readout, these fluorogenic assays enable the correct screening of activity modulators.

## Figures and Tables

**Table 1:** The fluorescence properties and SIRT1/2 activities of chromenone compounds.

Compound	Structure	Fluorescence			Activity (10 μM)	
		*E*_x_	*E*_m_	φ	SIRT1	SIRT2
**1**	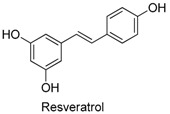	304	400	0.16	11.60	0.87
**2**	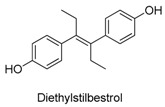	-	-	-	0.76	0.72
**3**	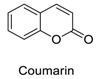	-	-	-	0.95	0.83
**4**	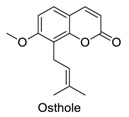	-	-	-	0.82	0.47
**5**	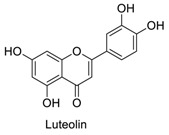	-	-	-	1.21	0.75
**6**	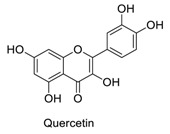	-	-	-	3.22	0.80
**7**	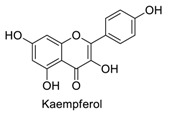	-	-	-	1.10	0.86
**8**	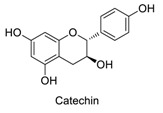	-	-	-	0.89	0.80
**9**	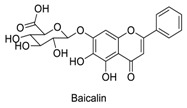	-	-	-	0.65	0.81
**10**	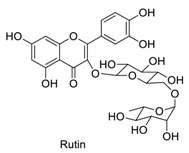	-	-	-	0.82	0.76
**11**	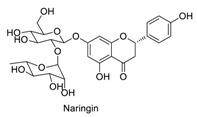	-	-	-	0.90	0.79
**12**	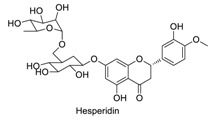	-	-	-	0.92	0.79
**13**	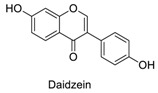	340	490	0.05	0.92	0.80
**14**	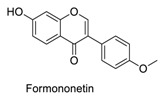	338	488	0.08	0.80	0.95
**15**	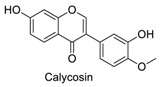	340	490	0.03	0.87	0.83
**16**	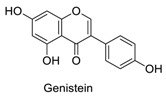	-	-	-	1.00	0.72
**17**	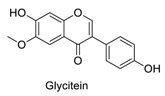	348	478	0.04	0.79	0.66
**18**	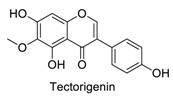	-	-	-	0.81	0.77
**19**	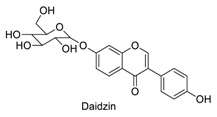	-	-	-	1.00	0.93
**20**	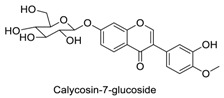	-	-	-	0.96	0.93
**21**	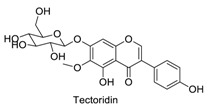	-	-	-	0.93	0.88

Compounds SRT1720 (S1129) and AGK2 (selleckchem, S7577) were used as the reference compounds to evaluate the SIRT1/2 assays.

## Supplementary Materials

Supplementary File 1
