# Supplementary material for: Exploration of the Fluorescent Properties and the Modulated Activities against Sirtuin Fluorogenic Assays of Chromenone-Derived Natural Products
Source: Molecules. 2018 May 2;23(5):1063. doi: 10.3390/molecules23051063 (PMC6100537; doi:10.3390/molecules23051063)

Exploration of the fluorescent properties and the modulated activities against sirtuin fluorogenic assays of chromenone-derived natural products

Hui Wen[1,†], Nina Xue[1,†], Feng Wu[2], Yujun He[1], Gang Zhang[1], Zebin Hu[3,*], and Huaqing Cui[1,*]

[1] State Key Laboratory of Bioactive Substances and Function of Natural Medicine, Institute of Materia Medica, Peking Union Medical College and Chinese Academy of Medical Sciences, Beijing 100050, China; wenhui@imm.ac.cn; angelnina@imm.ac.cn; heyujun506@imm.ac.cn; gzhang@imm.ac.cn
[2] Institute of Aviation Medicine, Air Force, Beijing, 100142, China; wufeng879@126.com
[3] National Institutes for Food and Drug Control, Beijing, 100050, China.
* Correspondence: H.C.: hcui@imm.ac.cn; Tel.: +86-10-63037952; Z.H.: huzbcmba@163.com; Tel.: +86-10-67095597;
[†] These authors contributed equally to this paper.

AMC

The excitation and emission fluorescence spectra of AMC: We set the Em = 450 nm to obtain the excitation fluorescence spectra (left), we set the Ex= 350 nm to obtain the emission fluorescence spectra (right).

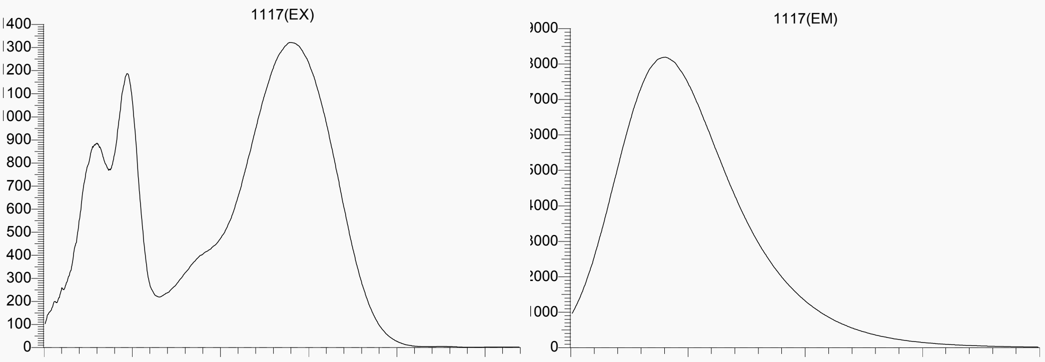

Resveratrol

*The excitation and emission fluorescence spectra of Resveratrol: We set the Em =**400** nm to obtain the excitation fluorescence spectra (left), we set the Ex=**304** nm to obtain the emission fluorescence spectra (right).*

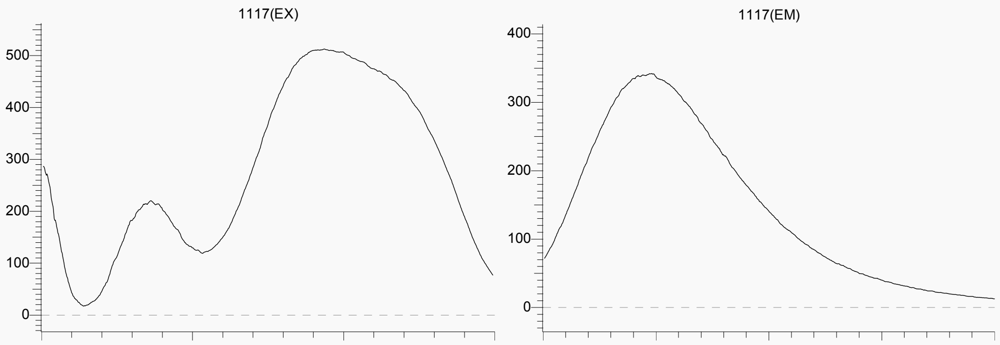

Daidzein

The excitation and emission fluorescence spectra of Daidzein: We set the Em = 490 nm to obtain the excitation fluorescence spectra (left), we set the Ex= 340 nm to obtain the emission fluorescence spectra (right).

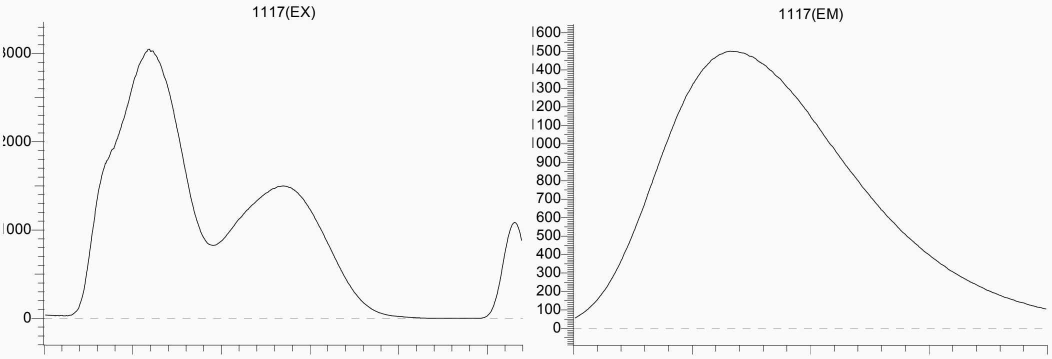

Glycitein

The excitation and emission fluorescence spectra of Glycitein: We set the Em =490 nm to obtain the excitation fluorescence spectra (left), we set the Ex= 340 nm to obtain the emission fluorescence spectra (right).

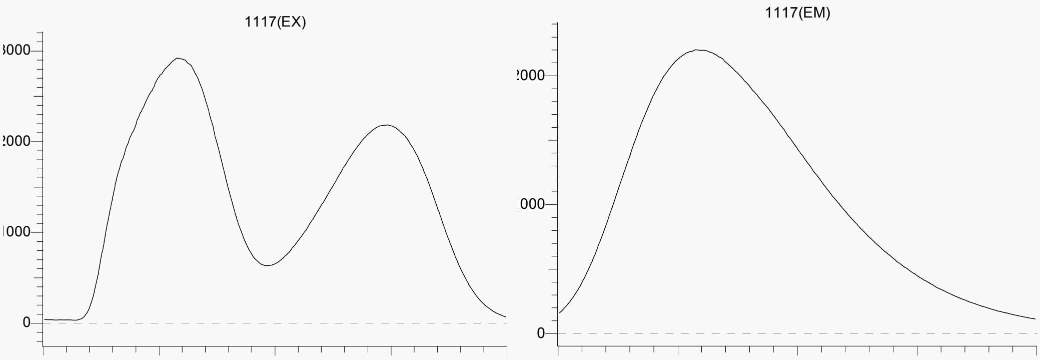

Formononetin

The excitation and emission fluorescence spectra of Formononetin: We set the Em =488 nm to obtain the excitation fluorescence spectra (left), we set the Ex= 338 nm to obtain the emission fluorescence spectra (right).

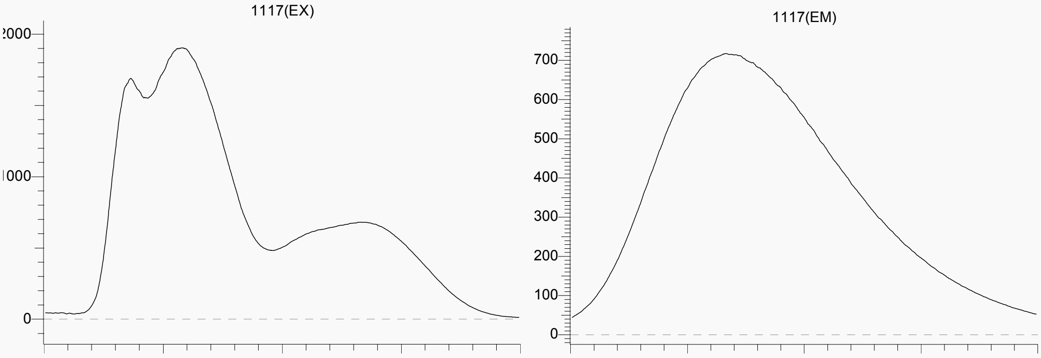

Calycosin

The excitation and emission fluorescence spectra of Calycosin: We set the Em =478 nm to obtain the excitation fluorescence spectra (left), we set the Ex= 348 nm to obtain the emission fluorescence spectra (right).

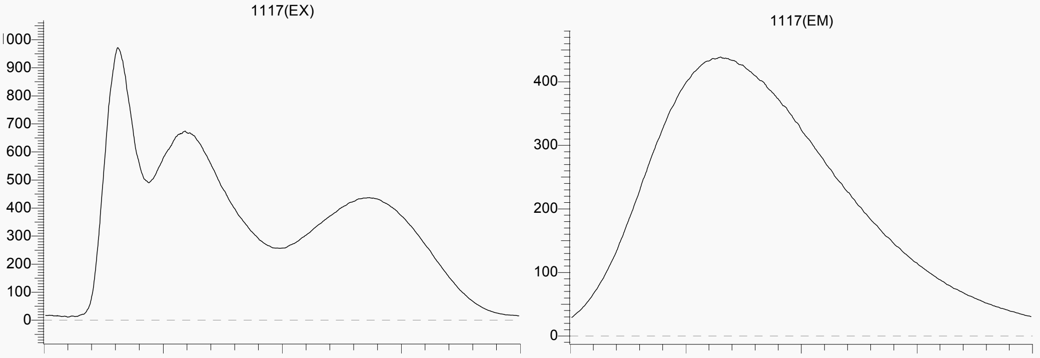

Supplement: Supplementary File 1 [file molecules-23-01063-s001.pdf]
